# Synchronous versus metachronous spinal metastasis: a comparative study of survival outcomes following neurosurgical treatment

**DOI:** 10.1007/s00432-024-05657-x

**Published:** 2024-03-19

**Authors:** Mohammed Banat, Anna-Laura Potthoff, Motaz Hamed, Valeri Borger, Jasmin E. Scorzin, Tim Lampmann, Harun Asoglu, Logman Khalafov, Frederic C. Schmeel, Daniel Paech, Alexander Radbruch, Louisa Nitsch, Johannes Weller, Ulrich Herrlinger, Marieta Toma, Gerrit H. Gielen, Hartmut Vatter, Matthias Schneider

**Affiliations:** 1 Department of Neurosurgery, University Hospital Bonn, Venusberg-Campus 1, Building 81, 53127 Bonn, Germany; 2 Department of Neuroradiology, University Hospital Bonn, Bonn, Germany; 3 Department of Neurology, University Hospital Bonn, 53127 Bonn, Germany; 4 Division of Clinical Neuro-Oncology, Department of Neurology, University Hospital Bonn, Bonn, Germany; 5 Institute of Pathology, University Hospital Bonn, Bonn, Germany; 6 Institute for Neuropathology, University Hospital Bonn, Bonn, Germany

**Keywords:** Surgery for spinal metastasis, Synchronous versus metachronous tumor occurrence, Survival, Neuro-oncology

## Abstract

**Purpose:**

Patients with spinal metastases (SM) from solid neoplasms typically exhibit progression to an advanced cancer stage. Such metastases can either develop concurrently with an existing cancer diagnosis (termed metachronous SM) or emerge as the initial indication of an undiagnosed malignancy (referred to as synchronous SM). The present study investigates the prognostic implications of synchronous compared to metachronous SM following surgical resection.

**Methods:**

From 2015 to 2020, a total of 211 individuals underwent surgical intervention for SM at our neuro-oncology facility. We conducted a survival analysis starting from the date of the neurosurgical procedure, comparing those diagnosed with synchronous SM against those with metachronous SM.

**Results:**

The predominant primary tumor types included lung cancer (23%), prostate cancer (21%), and breast cancer (11.3%). Of the participants, 97 (46%) had synchronous SM, while 114 (54%) had metachronous SM. The median overall survival post-surgery for those with synchronous SM was 13.5 months (95% confidence interval (CI) 6.1–15.8) compared to 13 months (95% CI 7.7–14.2) for those with metachronous SM (*p* = 0.74).

**Conclusions:**

Our findings suggest that the timing of SM diagnosis (synchronous versus metachronous) does not significantly affect survival outcomes following neurosurgical treatment for SM. These results support the consideration of neurosurgical procedures regardless of the temporal pattern of SM manifestation.

## Introduction

Systemic tumor disease with singular or multiple spinal metastases (SM) has assumed an increasingly prominent role in the daily clinical practice of spine surgeons and the lives of affected patients (Coleman 2006; Brande et al. 2022). It is estimated that approximately 5–15% of all cancer patients will ultimately develop spinal metastases (Brande et al. 2022; Jenis et al. 1999; Jacobs and Perrin 2001). Among the primary culprits are breast cancer, prostate cancer, and lung cancer, with the primary tumor remaining elusive in 3–10% of cases (Greenlee et al. 2000; Ulmar et al. 2007).

In the therapeutic arsenal for this profoundly affected patient population, surgery stands as a common treatment modality (Furlan et al. 2022). After lungs and liver, skeletal system and bones bear the brunt of systemic metastases (Macedo et al. 2017; Maccauro et al. 2011). Surgical options for managing spinal metastases encompass a spectrum, from biopsy coupled with vertebroplasty or kyphoplasty (Stangenberg et al. 2017; Georgy 2010), to spinal canal decompression in isolation (Patchell et al. 2005), or in conjunction with minimally invasive percutaneous procedures (Miscusi et al. 2015) and open instrumentation with augmented screws (Ringel et al. 2017; Park et al. 2019a), at times necessitating anterior–posterior stabilization (Ulmar et al. 2006; Gezercan et al. 2016). The overarching objective of surgical intervention is the mitigation or prevention of neurological deficits, coupled with a focus on enhancing the patient’s quality of life (Fehlings et al. 2016; Depreitere et al. 2020). Additionally, surgery provides a means to attain a definitive histological diagnosis of the spinal tumor lesion and potentially improves overall survival (OS) (Patchell et al. 2005; Krober et al. 2004).

SM may arise within the context of a previously known and managed systemic cancer disease (metachronous presentation), often preceded by multimodal therapies, such as radiation, systemic chemotherapy, immunotherapy, or specifically targeted therapies (Gerszten et al. 2009; Berger 2008; Choi et al. 2015, 2019). Alternatively, newly diagnosed SM may serve as the inaugural presentation of a previously undiscovered systemically disseminated cancer (synchronous presentation) (Jacobs and Perrin 2001; Bollen et al. 2018; Patnaik et al. 2020).

Despite existing literature, it remains uncertain whether the choice to surgically resect SM in cases of synchronous versus metachronous presentation significantly influences surgical decisions and patient survival. This study seeks to clarify this issue by examining the prognostic implications of synchronous versus metachronous SM diagnoses, measured from the day of neurosurgical SM resection, in patients who underwent surgical intervention for SM.

## Methods

### Patients and inclusion criteria

This study is based on consecutive patients aged >18 years who had undergone primary spinal canal decompression, with or without instrumentation, for SM between 2015 and 2020 at the neurosurgical department of the University Hospital Bonn. Comprehensive clinical data, including age, gender, primary tumor type, SM location, details of the neurosurgical procedure, the extent of spinal vertebrae involvement, American Society of Anesthesiologists (ASA) score, clinical-neurological assessment, and functional status measured by the American Spinal Injury Association (ASIA) Score (2019), were recorded.

Functional status was further evaluated using the Karnofsky Performance Scale (KPS) upon admission, categorizing patients into KPS ≥ 70% or KPS < 70%, as previously described (Schuss et al. 2021; Hamed et al. 2023; Schweppe et al. 2023; Ilic et al. 2021). The Charlson Comorbidity Index (CCI) was employed to quantify the comorbidity burden of patients before undergoing surgery (Hamed et al. 2022a; Schneider et al. 2020; Lehmann et al. 2023).

Overall survival (OS) was calculated from the date of surgical SM resection until death as previously described (Hamed et al. 2022b). Patients for whom no further follow-up information regarding survival was obtainable, typically due to ongoing treatment at external healthcare institutions, were excluded from subsequent statistical survival analysis.

Following histopathological analysis, all patients underwent thorough assessment by our internal Neurooncological Tumor Board, comprised of neurosurgeons, radiation therapists, neurooncologists, and neuroradiologist. Recommendations for post-surgery management were established through interdisciplinary consensus, occasionally coordinated with the treatment plans of referring physicians (Schafer et al. 2021).

Patients were categorized into two distinct cohorts for further analysis: those with SM diagnosed as a manifestation of a previously known cancer (metachronous presentation) and those with a new diagnosis of SM as the initial indication of an undiscovered cancer (synchronous presentation) (Potthoff et al. 2023).

Exclusion criteria encompassed patients classified as non-operable and those lacking complete data or follow-up information. Pertinent clinical parameters, including preoperative functional neurological status, comorbidities, radiological characteristics, primary cancer site, and the timing of diagnosis, were assessed for analysis.

The study adhered to the ethical principles outlined in the 1964 Helsinki Declaration and received approval from the Ethics Committee of the University Hospital Bonn (protocol no. 067/21). Given the retrospective nature of the study, the acquisition of informed consent from participants was not pursued.

### Statistical analysis and graphical illustration

Data collection and analysis were conducted utilizing the SPSS computer software package for Windows (Version 27, IBM Corp., Armonk, NY). Categorical variables underwent analysis through contingency tables, employing the Fisher’s exact test when assessing two variables and the chi-square test when evaluating more than two variables. Non-normally distributed data were subjected to the Mann–Whitney *U* test. Overall survival (OS) rates were assessed using the Kaplan–Meier method, with Graph Pad Prism software for MacOS (Version 9.4.1, Graph pad Software, Inc., San Diego, California, USA) employed for this purpose. Survival rate comparisons were performed utilizing the Gehan–Breslow–Wilcoxon test. To identify predictors of elevated 1-year mortality, a multivariate logistic regression model was constructed using a backward stepwise approach. Statistical significance was determined at *p* < 0.05. Furthermore, the radar plot was generated using R (Version 3.6.2, Vienna, Austria), as previously outlined in reference (Lehmann et al. 2021).

## Results

### Patient and tumor characteristics

Between 2015 and 2020, 211 patients had undergone resection of SM at the Neurosurgical Department of the University Hospital Bonn. The median patient age at the day of surgery was 66 years (interquartile range (IQR) 57–74 years) (Table 1). The most common primary tumor site was the lung (23%), followed by the prostate (22%) and the breast (11%). The thoracic spine was the most commonly affected segment of the spine with 56%. Single or dual-level disease was present in 126 patients (60%), whereas multilevel infiltration was present in 85 patients (40%). The majority of patients (62%) underwent decompression and dorsal stabilization, while spinal canal decompression alone was performed in 38% of the patients. Median CCI of the entire patient cohort was 8 (IQR 6–10). 67% of our cohort presented with a preoperative KPS score of ≥ 70. Median OS for the entire study cohort with surgically treated SM was 13 months (IQR 3–23).

**Table 1:** Patient characteristics

	*n* = 211
Median age (IQR) (in yrs)	66 (57–74)
Female sex	81 (38.5)
Primary tumor site	
Lung	49 (23.0)
Breast	24 (11.4)
Prostate	46 (22.0)
Others	92 (43.6)
Location of disease	
Cervical	21 (10.0)
Thoracic	118 (56.0)
Lumbar	36 (17.0)
Combined	36 (17.0)
Surgery	
Decompression	81 (38)
Stabilization	130 (62)
Levels of disease	
1–2	126 (60)
≥3	85 (40)
Median CCI (IQR)	8 (6–10)
KPS ≥ 70	141 (54)
Pre-operative neurological deficit (ASIA A-C)	66 (31)
Median OS (IQR) (in months)	13 (3–23)
Time of SM diagnosis	
Synchronous	97 (46)
Metachronous	114 (54)

Values represent the number of patients unless indicated otherwise (%)

*ASA* American Society of Anesthesiology physical status classification system, *ASIA* American Spinal Injury Association, *CCI* Charlson Comorbidity Index, *KPS* Karnofsky Performance Scale, *IQR* interquartile range, *n* number of patients, *OS* overall survival, *SM* spinal metastasis, *yrs* years

79 of 211 of patients (46%) suffered from synchronous SM, 114 of 211 patients (54%) exhibited metachronous SM. For further more details of patient- and tumor-related characteristics, see Table 1.

### Survival rates do not significantly differ between synchronous and metachronous spinal metastases

**Fig. 1:**
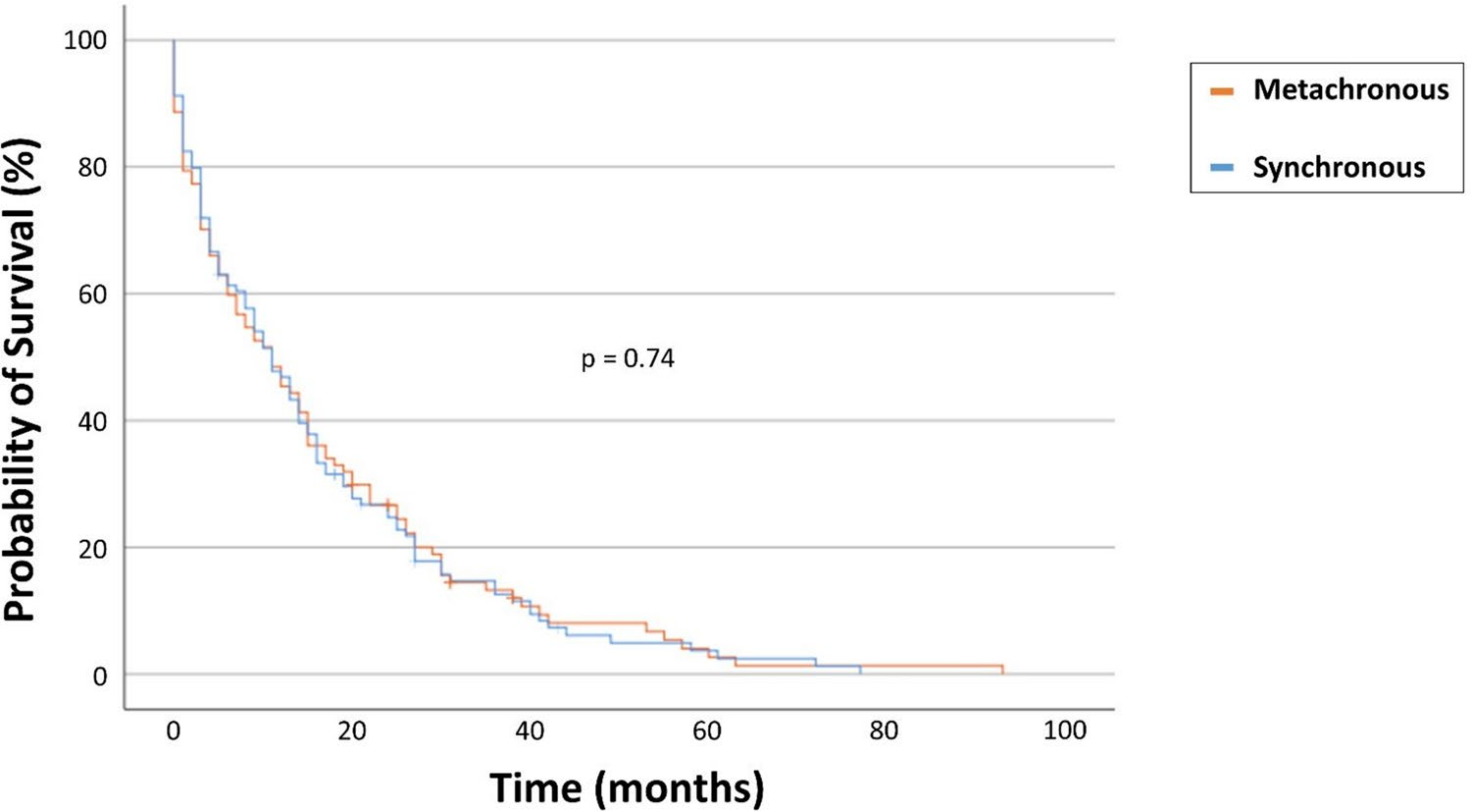
Kaplan–Meier survival analysis dependent on synchronous vs. metachronous SM occurrence. SM, spinal metastasis; vs., versus

In the synchronous SM group, 50 out of 93 patients (54%) succumbed within 1 year following surgical resection, compared to 60 out of 107 patients (56%) in the metachronous SM group (*p* = 0.78) (Table 2). The mOS for patients with synchronous SM diagnosis was 13.5 months (95% CI 6.1–15.8), while patients with metachronous SM diagnosis exhibited a mOS of 13.0 months (95% CI 7.7–14.2) when calculated from the day of SM surgical treatment (*p* = 0.74) (Fig. 1).

**Table 2:** Patients with surgically treated SM stratified for synchronous vs. metachronous SM occurrence

	Synchronous diagnosis of SM *n* = 97	Metachronous diagnosis of SM *n* = 114	*p* value
Median age (yrs)	67 (58–73)	65 (57–75)	0.8
Female sex	46 (47)	35 (31)	**0.02**
Pre-operative KPS < 70	27 (28)	43 (38)	0.1
Primary tumor site			
Lung	34 (35)	15 (4)	**<0.001**
Breast	20 (21)	4 (4)	**<0.001**
Prostate	14 (14)	32 (28)	**0.019**
Other	29 (30)	63 (55)	**<0.001**
Location of disease			
Cervical	12 (12)	9 (8)	0.4
Thoracic	50 (52)	68 (60)	0.28
Lumbar	15 (15)	21 (18)	0.35
Combined	20 (21)	16 (14)	0.27
Levels of disease			0.2
1–2	53 (55)	73 (64)	
≥3	44 (45)	41 (36)	
Median CCI (IQR)	8 (7–10)	8 (6–10)	0.7
Preoperative neurological deficit (ASIA A-C)	33 (34)	33 (29)	0.5
Surgery			0.05
Decompression	31 (32)	50 (44)	
Stabilization	66 (68)	64 (56)	
1-year mortality	50/93* (54)	60/107** (56)	0.78
Median OS (95% CI)	13.5 (6.1–15.8)	13.0 (7.7–14.2)	0.74

Values represent the number of patients unless indicated otherwise (%)

The values in bold are statistically significant

*CCI* Charlson Comorbidity Index, *IQR* interquartile range, *OS* overall survival, *SM* spinal metastasis, *yrs* years

* 4 of 97 patients censored with lost to follow-up <12 months

** Median (IQR). 7 of 114 patients censored with lost to follow-up **<**12 months

**Fig. 2:**
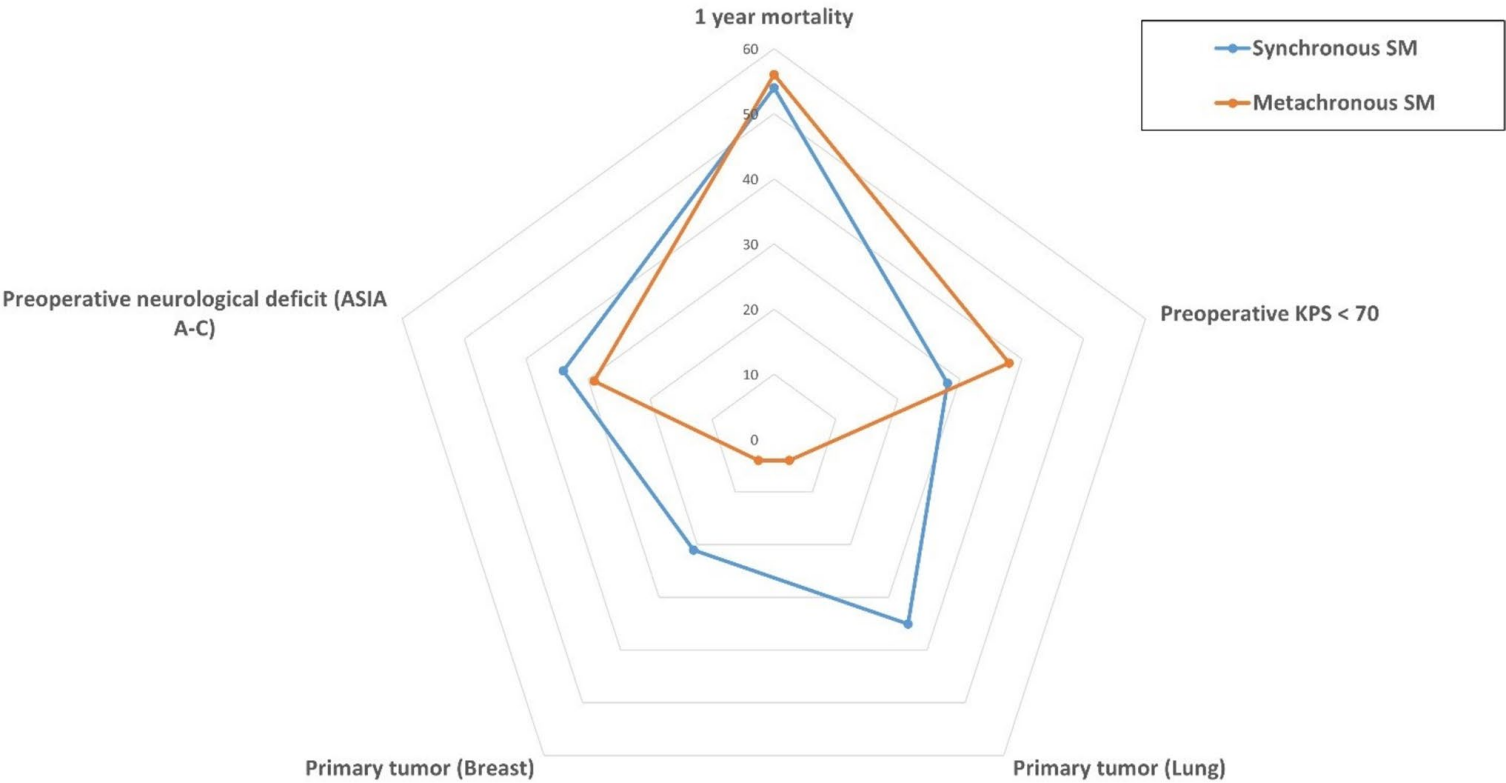
Radar plot depicting patient- and disease-related characteristics dependent on synchronous vs. metachronous SM occurrence in patients with surgically treated SM. CCI, Charlson comorbidity index; KPS, Karnofsky performance score; mOS, median overall survival; SM, spinal metastasis; vs., versus

Lung and breast carcinomas were significantly more common in the synchronous group, whereas prostate carcinoma was the most common tumor entity in the metachronous group (Table 2). The female gender was also significantly more frequently affected in the synchronous situation, with breast carcinoma being included. All the other parameters included in Table 2 did not significantly differ between the groups of synchronous and metachronous SM (Fig. 2; Table 2).

### Multivariable analysis for predictors of 1-year mortality

We performed a multivariable regression analysis including the variables sex, preoperative KPS, preoperative CCI, tumor entity, and time of diagnosis (synchronous versus (vs.) metachronous) in order to identify independent predictors of 1-year mortality following surgery for SM.

The multivariable analysis revealed preoperative KPS < 70 (OR 0.1, 95% CI 0.06–0.2, *p* < 0.001), preoperative CCI > 10 (OR 0.5, 95% CI 0.2–0.9, *p* < 0.001), and tumor entity breast (OR 0.2, 95% CI 0.07–0.7, *p* = 0.01) as significant and independent predictors of 1-year mortality (Table 3). Time of SM diagnosis (synchronous vs. metachronous SM presentation) did not meet statistical significance (OR 0.7, 95% CI 0.4–1.4, *p* = 0.3).

**Table 3:** Multivariable regression analysis for predictors of 1-year mortality

Factors	Adjusted OR	95% CI	*p* value
Female sex	0.6	0.3–1.2	0.2
Pre-operative KPS < 70	9.8	4.6–20.7	**<0.001**
CCI > 10	0.5	0.2–0.9	**0.03**
Tumor entity			
Lung	1.7	0.8–3.6	0.2
Breast	0.2	0.07–0.7	**0.01**
Synchronous diagnosis of SM	0.7	0.4–1.4	0.3

The values in bold are statistically significant

*CCI* Charlson Comorbidity Index, *CI* confidence interval, *KPS* Karnofsky Performance Scale, *OR* odds ratio, *SM* spinal metastasis

## Discussion

This study analyzes the prognostic impact of metachronous vs. synchronous SM diagnosis in patients who had undergone surgical therapy for SM. We found that the time of SM diagnosis does not impact 1-year mortality and patient survival when measured from the day of SM resection.

In the group of patients with SM from lung and breast cancer, SM significantly more often occurred in the synchronous than in the metachronous situation. Compared with this, SM from prostate and other carcinoma significantly more often occurred in the course of the known underlying cancer disease (metachronous situation). Lung cancer is notably associated with the highest incidence of spinal metastases (SM) and brain metastases (BM). The occurrence of SM in lung cancer patients, as reported in the literature, ranges from 5% to a significant 56%. This variation is influenced by factors, such as the histological type of the cancer, the status of the epidermal growth factor receptor (EGFR) mutation, and the stage of the disease (Berghoff et al. 2016; Nayak et al. 2012; Goncalves et al. 2016; Wang et al. 2017; Zhang et al. 2020; Rizzoli et al. 2013). Similarly, SM is observed in 5–15% of breast and prostate cancer cases, making these two types of cancer among the most common to develop SM (Rizzoli et al. 2013; Hong et al. 2020; Kumar et al. 2020; Park et al. 2019b). The observed difference in the frequency of synchronous versus metachronous spinal metastasis (SM) diagnosis between lung and prostate cancer may be partially attributed to the diagnostic practices for these cancers. Prostate cancer may often be detected during routine medical check-ups for men, leading to earlier diagnosis. In contrast, lung cancer typically remains undetected until it reaches more advanced stages of the disease (Goldsmith 2014; Lux et al. 2019; Vinas et al. 2016).

Our findings regarding the distribution of cancer entities align with those reported in well-established studies (Krober et al. 2004; Hosono et al. 2005; Sciubba and Gokaslan 2006). Consistent with numerous publications, we observed that the thoracic spine was the most frequently affected spinal segment in both synchronous and metachronous SM groups (Bach et al. 1990; Comey et al. 1997). However, our study did not identify a specific dissemination pattern linked to the primary tumor, such as a preference for lung cancer metastases to manifest singularly or multiply in the thoracic spine, as noted in some reports (Schiff et al. 1998; Gilbert et al. 1978). Conversely, other researchers have observed a concentration of bronchial carcinoma in the thoracic spine and a predominance of prostate carcinoma in the lumbar spine (Krober et al. 2004).

In contemporary literature, the incidence of multiple spinal canal metastases in cases of spinal infiltration with SM is reported to be up to 30% (Sande et al. 1990). Our cohort demonstrates a prevalence with 45% in synchronous SM and 36% in metachronous SM involving more than three segments.

To the best of our knowledge, this study is the first to investigate the prognostic impact of synchronous versus metachronous SM. A notable aspect of our approach is the emphasis on postoperative survival in the survival analysis. This focus is crucial as it aligns with the typical juncture at which neurosurgeons encounter patients with spinal metastasis. These findings suggest that the indication for surgery should be considered regardless of whether the SM is synchronous or metachronous. This conclusion is significant for clinical decision-making in neurosurgery, suggesting that the timing of metastasis, in relation to the primary tumor, should not be a deterrent to surgical intervention.

In essence, our findings advocate for a surgical approach in managing spinal metastasis without bias toward the metastasis’ temporal classification. This has direct implications for neurosurgical management, underscoring the importance of considering surgery as a viable treatment option in both synchronous and metachronous scenarios and providing a clear directive for surgical intervention.

### Limitations

This study is subject to a number of limitations. First, the data collection was retrospective in nature, and there was no randomization of patients; instead, treatment decisions were made based on the individual preferences of physicians at our institution. Additionally, the study population of patients with SM is notably diverse, encompassing a range of underlying cancer types and varying pre-treatment histories. Despite these limitations, our findings might provide a basis for the establishment of multicenter registries and the development of further prospective studies.

## Conclusions

The present study indicates that the timing of SM diagnosis, whether synchronous or metachronous, does not substantially influence patient survival following surgical treatment. These findings imply that decisions regarding neurosurgical intervention should be considered independently of the temporal classification of SM.

## Data Availability

The datasets generated during and/or analysed during the current study are available from the corresponding author on reasonable request.

## Abbreviations

ASA: American Society of Anesthesiologists
ASIA: American Spinal Injury Association
CCI: Charlson Comorbidity Index
CI: Confidence interval
KPS: Karnofsky Performance Scale
SM: Spinal metastases
OS: Overall survival
